# Sats can be used for mortality prediction

**DOI:** 10.1186/2197-425X-3-S1-A966

**Published:** 2015-10-01

**Authors:** J Massaut, O Chery, G Suy, L Pierre Louis, P Valles

**Affiliations:** ULB, Bruxelles, Belgium; Hopital Nap Kenbe, Anaesthesia Intensive Care, Port au Prince, Haiti; Hopital Nap Kenbe, Emergency, Port au Prince, Haiti; Médecins Sans Frontières, Sage Unit, Brussels, Belgium

## Introduction

The South African Triage Scale (SATS) [1] was developed to facilitate triage of patients in emergency departments. It is used by Médecins Sans Frontières (MSF) in several centers. Predicting the risk of death in a health institution is also important and can be used as a tool for quality management.

## Objectives

The aim of this study was to determine if SATS can be used to predict hospital mortality, and to use the results to follow mortality in a Emergency and Trauma Center of MSF.

## Methods

We did not find any publication reporting mortality prediction using SATS. To determine mortality prediction, we decided to use data from patients admitted to the Hopital Nap Kenbe of MSF in Port au Prince, Haiti. Data from 5813 patients admitted to that institution from 01/01/2013 to 31/12/2014 were used. Age of the patients, motif of admission according to MSF classification, triage category (Red, Orange, Yellow, Green) according to the SATS system were extracted from the local MSF data base and used as candidate parameters for analysis of factors associated with mortality. For the statistical analysis we used Chi Square and Mann-Whitney test to determine association with mortality and logistic regression and logit to determine independent factors, compute odd ratios and predict mortality. P values < 0.05 were considered as significant. For logistic regression we divided patients in two groups at random. The first group was used for the computations of Odds ratios and coefficients, the second for validation of the model. We performed post-hoc analysis to compare observed with predicted mortality, dividing the time scale in trimesters. The STATA 8 software was used for the analysis.

## Results

Hospital mortality varied significantly with age of the patients, motif of admission and triage category. Odds ratios for factors affecting mortality with coefficients of logit estimates for the model are illustrated in table 1.

**Table 1:** Factors affecting Mortality.

Factors	Odds Ratios	Coefficients	Z	P > z
Age > 45	2.681	0.986	2.88	0.004
Red	80.139	4.383	2.35	0.019
Orange	3.969	1.378	2.96	0.003
Non Trauma	4.159	1.425	2.88	0.004
(Constant)		-6.449	-12.16	0.000

The surface under the ROC curve was 0.869 for the first group and 0.860 for the validation group, where 98.14% of the patients were correctly classified.

**Figure 1:**
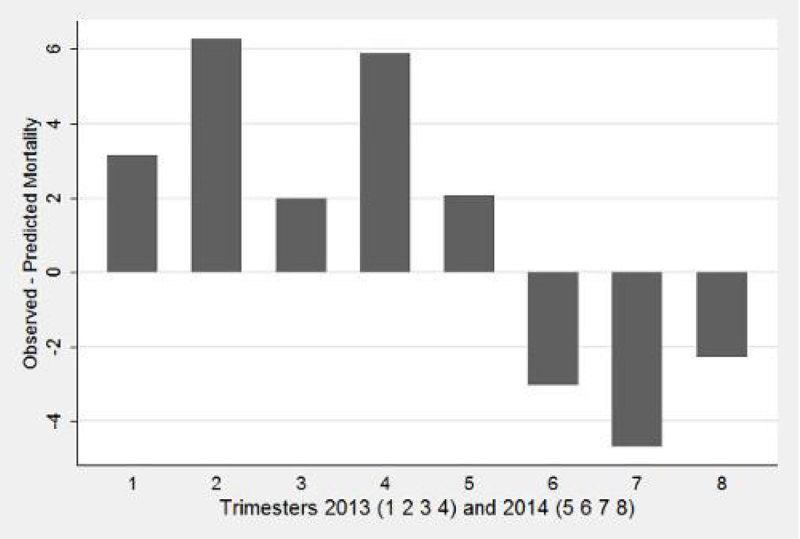
Figure 1

Observed mortality exceeds predicted mortality in 2013. The reversed was observed for the 3 last trimesters of 2014 and seems to reveal a learning process leading to better quality with time, as illustrated by figure 1.

## Conclusions

Our work indicates that SATS with age and motif of admission can be used to predict and follow hospital mortality. It must be validated in a pluri-center study before to be used elsewhere.
